# Factors Associated with Acculturative Stress among International Medical Students

**DOI:** 10.1155/2020/2564725

**Published:** 2020-06-21

**Authors:** Magdalena Iorga, Camelia Soponaru, Iulia-Diana Muraru, Sofia Socolov, Florin-Dumitru Petrariu

**Affiliations:** ^1^Faculty of Medicine, University of Medicine and Pharmacy “Grigore T. Popa”, Iasi, Romania; ^2^Faculty of Psychology and Educational Sciences, University “Alexandru Ioan Cuza”, Iasi, Romania

## Abstract

There is an array of reasons why acculturation can be stressful, and acculturative stress can be triggered by a plethora of factors. The aim of the study was to identify factors associated with acculturative stress among international students enrolled in a public medical university from Romania. 265 students were included in the research. Sociodemographic, academic, and family data, comfortability with living in study city, satisfaction with administrative staff, colleagues, and professors and Acculturative Stress Scale for International Students were gathered. Data have been processed using SPSS Statistics v23.0.0 for MAC.OSX. Female students are more prone to experience homesickness and stress due to change compared to male students. International students with Romanian origins had lower scores on perceived hate and stress due to change/culture shock compared to those with no Romanian origins. Students with relatives or friends enrolled in the same university had significantly lower levels of acculturative stress, perceived discrimination, perceived hate/rejection, and general/nonspecific concerns. Age and year of study are associated with homesickness and stress due to change/culture shock. The existence of factors associated with acculturative stress demand institutional, social, and psychological support for international students.

## 1. Introduction

Universities all over the world accommodate a large number of international students. In recent years, Romania, as many other Eastern European countries, also attracted many international students who enrolled especially in medical studies. The main reasons for studying in Romania are cheaper academic taxes, affordable accommodation and transport, peaceful university city, religious tolerance, and a European country [1, 2]. However, the choice to study abroad has a substantial impact on the student and the acculturation process can lead to acculturative stress and difficulties adjusting to the environment of the host country.

Acculturation is the process of both cultural and psychological change that occurs when two or more cultural groups (along with their individual members) come into contact [3]. It involves changes on two major levels: the group level (it entails changes in social structures and institutions and in cultural practices) and the individual level (it comprises the change of an individual's behaviors). All these changes are the result of a long-term process that should not be conceptualized as unidirectional, in that immigrants assimilate into their adopted country, but the host culture does not change [4]. In the specific context of academic relationship, teachers working with international students must adapt themselves to multicultural and multilingual groups and must adjust their teaching styles to diminish the barriers related to language, cultural, ethnicity, etc.

Given the fact that acculturation can be perceived as a stressful experience, the term acculturative stress is generally used to describe the unique stressors of immigration [5]. In other words, the experience of acculturation is a series of major life events that can be challenging for an individual and can trigger a stress reaction called acculturative stress [6]. Ward and Geeraert [7] believe that acculturation demands facing acculturative stressors coupled with the acquisition, maintenance, and change of cultural attitudes, behaviors, values, and identities. There are several factors that may contribute to acculturative stress, including the way newcomers are regarded by members of the receiving society [8], the motivation for immigrating [9], and the separation of families [10].

In the case of college students, they already are a population prone to experience higher levels of stress and depression [11–13] and the reasons for this include demanding tasks, very busy university schedule, close deadlines, living away from families, financial problems, difficulties having regular meals, and eating unhealthy foods [14–16]. Among them, medical students stand out. Their academic education is the longest, and they are often faced with numerous uncomfortable situations (communicating with ill individuals, facing palliative care patients, exploring doctor-patient-family relationship, or experiencing life-ending situations). The specificity of medical studies adds pressure and stress, and preexisting patterns established during the students' years of training can continue into adult life.

International students represent an important contribution to the intellectual capital of their host country and to the workforce by bringing with them a variety of knowledge and skills in many areas [17]. They bring forth cultural exchange and understanding and create a more diverse learning environment [18]. Still, although the presence of international students in the host country is mutually beneficial, there are numerous factors impacting upon international students' acculturation process and they may experience acculturative stress and adjustment problems. Furthermore, acculturative stress has several consequences on the mental health of college students: depression [19], bulimic symptoms [20], and body image disturbance [21]. Therefore, understanding the factors associated with acculturative stress in this specific population is important for universities and mental health care professionals alike.

Within the international student literature, there are some frequently documented acculturative stressors. One of the most well-documented such stressors is the language barrier. English fluency is a good predictor of acculturative stress [22], and English competency is a predictor of adjustment [23] in international students. Also, although academic stress is not unique to international students, it is likely to be more intense in their case. The reasons include the added stressors of second language anxiety and adapting to a new educational environment [17].

Sociocultural stressors represent another challenge for international students. In a study conducted by Zhang and Brunton [24], more than half (55%) of the international students in their sample were unhappy with their opportunities to make friendships with locals and 71% expressed the desire to have more local friends. Also, Sawir et al. [25] found that 65% of the participants in their study reported that they had experienced periods of loneliness and/or isolation.

When studying in another country, students might also experience practical or lifestyle acculturative stressors such as financial difficulties, accommodation, and transportation [26]. Also, international students' dietary habits change [27, 28] and they experience emotional and physical support when consuming home country food [29].

The research on students' acculturative stress is not yet conclusive, especially from the point of view of medical studies. Clarity is essential to ensure that appropriate interventions are introduced which will positively impact international medical students and research on this area needs to extensively identify factors that can influence acculturative stress. The aim of the study was to evaluate the presence of acculturative stress among international students from a medical university in Romania and to identify associated factors. We addressed three main categories of factors: sociodemographic (age; sex; having relatives, friends or siblings enrolled in the same university; if parents are doctors; etc.), comfortability with various aspects of living in the study city (speaking English and Romanian, neighbors, climate, transportation, and food), and satisfaction with administrative staff, colleagues, and professors. The present research is part of a larger study and previous results revealed that climate and food appeared to be the most uncomfortable aspects that students must deal with [2].

## 2. Materials and Methods

### 2.1. Participants

A number of 300 questionnaires were distributed among international medical students enrolled in General Medicine and Medical Dentistry faculties. The Medical University is located in the northeast part of Romania, specifically in the city of Iasi. They were invited to participate voluntarily in the research. They had to provide information concerning sociodemographic, academic, and family data. Several items were constructed especially for the research, in order to identify the level of student's comfortability with living in the university city and the level of satisfaction with administrative staff, colleagues, and professors. The third part of the data sheet measured the acculturative stress by using a psychological instrument.

Students were informed about the purpose of the study and confidentiality of collected data; withdrawal was accepted at any time. A total of 289 questionnaires were returned to the researchers. For statistical analysis, 265 were finally considered; 24 questionnaires were eliminated for failing to complete at least 90% of the items.

### 2.2. Instruments

#### 2.2.1. Sociodemographic Data

Various sociodemographic, academic, and family data were registered: age, sex, year of study, nationality, if parents are doctors, if the student speaks Romanian, if parents speak Romanian, if at least one parent is a doctor, if parents visit every year, if the student is satisfied with living conditions, and having relatives, friends, or siblings enrolled in the university.

#### 2.2.2. Comfortability of Living in the Study City

The degree of comfort with living in the study city was measured using a self-rated scale. Participants responded using a Likert-type scale from 1 to 5 (1—not at all comfortable, 2—somewhat comfortable, 3—comfortable, 4—very comfortable, and 5—extremely comfortable). The items referred to their comfort with communicating in English, Romanian, and with neighbors. We also inquired about comfortability with climate, transportation, and food.

#### 2.2.3. Satisfaction with Colleagues/Staff/Teachers

To assess the satisfaction with administrative staff, colleagues, and teachers, several items were formulated, and responses were rated on a scale from 1 (very dissatisfied) to 10 (very satisfied).

#### 2.2.4. Acculturative Stress

ASSIS (The Acculturative Stress Scale for International Students) is a tool developed by Sandhu and Asrabadi in 1994 [30]. The instrument is composed of 36 items in the form of statements about the stress caused by the adaptation to a new culture, which participants evaluate using a Likert scale from 1 (strong disagreement) to 5 (strong agreement), depending on how much it characterizes them. The measured dimensions are as follows:

- perceived discrimination (8 items; “I am treated differently in social situations”)
- homesickness (4 items; “Homesickness for my country bothers me”)
- perceived hate/rejection (5 items; “I feel rejected when people are sarcastic toward my cultural values”)
- fear (4 items; “I fear for my personal safety because of my different cultural background”)
- stress due to change/culture shock (3 items; “I feel uncomfortable to adjust to new cultural values”)
- guilt (2 items; “I feel guilty to leave my family and friends behind”)
- nonspecific concerns (10 items; “I feel nervous to communicate in English”)

The total scores range from 36 to 180, higher scores suggesting higher levels of stress. For the present study, the alpha Cronbach coefficient for the entire instrument is *α* = 0.96. Several studies focusing on international students reported adequate psychometric properties for this measure of acculturative stress obtaining an internal consistency coefficient of 0.92 or above [31, 32].

### 2.3. Ethical Approval

The study was conducted in accordance with the Declaration of Helsinki, and the protocol was approved by Centre de Reussite Universitaire, under the coordination of University of Medicine and Pharmacy and Agence Universitaire pour la Francophonie (AUF).

### 2.4. Statistical Analysis

Collected data were analyzed using IBM SPSS Statistics, version 23. Percentages, means, and standard deviations were used for the descriptive analysis. Pearson (for quantitative variables) and Spearman (for analyses involving ordinal variables) correlations were used to investigate the associations between variables. The independent samples *t*-test was used in the case of variables where there are two independent groups to determine if there are any statistically significant differences between the means of these groups. Given the high number of independent samples *t*-tests employed, the Benjamini-Hochberg procedure was used to decrease the false discovery rate. Using a false discovery rate of 25%, the procedure indicated that all our *p* values were significant (available here).

## 3. Results

### 3.1. Descriptive Analysis

265 students from 34 countries took part in the research (four students have dual nationality). The distribution according to gender is reasonably homogenous: 151 male (57%) and 114 females (43%). The participants were aged from 17 to 40 (*M* = 21.38, *SD* = 3.32).

Participants were from all years of study (first to sixth). Students were asked if they had friends, relatives, or colleagues among international students registered in the same university. Collected data showed that, of the students who reported having relatives/friends attending the same university, 33.9% of the them have brothers/sisters, 32.1% have cousins, 29.4% have other friends, and 4.6% other relatives. Data regarding Romanian origins (at least one parent is Romanian but migrated to another country), if at least one parent is a physician, if the student/mother/father speaks Romanian, if the parents visit them every year, and if students are satisfied with their living conditions are presented in Table 1.

The level of comfortability of international students is presented in Table 2. In the extremely comfortable category, the highest score was for speaking English (31.3%) and the lowest was for city transportation (3.0%). In the not comfortable category, the highest score was for speaking Romanian (35.8%) and the lowest was for speaking English (2.6%).

Mean levels of satisfaction with colleagues, teachers, and administrative staff were as follows: 7.18 ± 2.13, 6.22 ± 2.32, and 5.52 ± 2.42, respectively.

The results for Acculturative Scale for International Students (total score for ASSIS and values for each subscale) are presented in Table 3; the highest scores were identified for nonspecific concerns and perceived discrimination, and the lowest scores for guilt, fear, and stress due to change/culture shock.

### 3.2. Comparative Analysis

When comparing male and female students on the total score of ASSIS and each of its dimensions, two statistically significant differences emerged concerning homesickness (*t* (263) = −5.532, *p* < 0.001) and stress due to change (*t* (263) = −2.105, *p* = 0.036). Female students were more prone to experience homesickness (*M* = 12.61) and stress due to change (*M* = 7.88) compared to male students (*M* = 10.39 and *M* = 7.25, respectively).

The fact that students had Romanian origins revealed two statistically significant differences on two subscales of the ASSIS: perceived hate (*t* (261) = −2.110, *p* = 0.036) and stress due to change/culture shock (*t* (261) = −2.984, *p* = .003). More specifically, students who had Romanian origins had lower scores on perceived hate (*M* = 10.91) and stress due to change/culture shock (*M* = 6.12) compared to those with no Romanian origins (*M* = 11.80 and *M* = 7.66, respectively).

The fact that one parent was a doctor revealed two differences between participants concerning homesickness (*t* (263) = −2.172, *p* = .031) and guilt (*t* (263) = −3.063, *p* = .003). More specifically, students whose parents were doctors experienced lower levels of homesickness (*M* = 10.91) and guilt (*M* = 4.45) compared to those whose parents had other jobs (*M* = 11.39 and *M* = 4.80, respectively).

When taking into account the existence of other relatives enrolled in the same university, the results showed several statistically significant differences between international students in terms of acculturative stress (*t* (263) = −2.062, *p* = 0.040), perceived discrimination (*t* (263) = −2.299, *p* = 0.022), perceived hate/rejection (*t* (263) = −2.345, *p* = 0.020), and nonspecific concerns (*t* (263) = −2.031, *p* = 0.043). Students with relatives enrolled in the same university had significantly lower levels of acculturative stress (*M* = 82.43), perceived discrimination (*M* = 17.98), perceived hate/rejection (*M* = 10.98), and nonspecific concerns (*M* = 21.82) compared to those who did not have relatives enrolled in the same university (*M* = 88.30, *M* = 19.66, *M* = 12.13, and *M* = 23.56, respectively).

Analyses also revealed significant differences in stress due to change/culture shock between students who spoke Romanian (*t* (263) = −4.578, *p* < 0.001) or having their mother (*t* (263) = −3.595, *p* < 0.000) or father speaking Romanian (*t* (263) = −2.358, *p* = .019). Specifically, they had lower scores on this subscale (*M* = 5.80, *M* = 5.92, and *M* = 6.63, respectively) compared to those who did not speak Romanian (*M* = 7.67) and neither did their mother (*M* = 7.69) or father (*M* = 7.66). Also, students whose mothers spoke Romanian experienced lower levels of acculturative stress (*M* = 76.61) and perceived hate (*M* = 10.07) than those whose mothers who did not speak Romanian (*M* = 86.82, *M* = 11.81, respectively) *t* (263) = −2.162, *p* = 0.032; *t* (263) = −2.134, *p* = 0.034, respectively.

Also, there was a difference between participants whose parents visited them every year compared to those who did not visit them as often on the homesickness subscale: *t* (263) = 2.377, *p* = 0.018. The former scored higher on this subscale (*M* = 12.02) compared to the latter (*M* = 10.98).

### 3.3. Correlation Analysis

The results of the correlation analysis revealed several significant and negative associations between the study variables and are presented in Table 4. However, the effect sizes ranged from small (*r* = −0.123) to moderate (*r* = −0.381).

The results of our study indicated that acculturative stress and perceived discrimination correlated negatively with the degree of comfort regarding: communication in English, climate, neighbors, communication with the homeowner and food in the host country; perceived discrimination correlated with local transport and acculturative stress was found to correlate with age.

The statistical analysis revealed that homesickness was significantly correlated with age, year of study, the degree of comfort communicating in English, and climate of the host country.

Perceived hate/rejection and fear were found to be correlated significantly and negatively with the degree of comfort with English communication, climate, neighbors, and food.

Correlational analyses also showed stress due to change/culture shock correlated negatively with age, year of study, and with the degree of comfort with communication in English, communication in Romanian, climate, neighbors, and food from Romania.

The results suggested that guilt correlated significantly with age, year of study, and with the degree of comfort with communication in English, climate, and food in Romania.

According to the results, general/nonspecific concerns correlated significantly with age and with the degree of comfort studied by the research (communication in English, climate, neighbors, communication with the homeowner, and food).

Finally, the degree of satisfaction with colleagues was negatively correlated with the ASSIS and all its subscales. The degrees of both satisfaction with administrative staff and with teachers were correlated negatively with the total score of ASSIS and with its subscales, except homesickness.

## 4. Discussion

Previous studies have shown that there are a considerable number of factors associated with acculturative stress. Some of these factors include personality, social inclusiveness, language barriers, and cultural differences. Gender, age, and language competence are the most well-documented [33–35].

The results of our study showed that women are more prone to higher levels of homesickness and stress due to change/culture shock; a fact that is congruent with other research. For example, Castillo et al. [36] identified that international female students had higher levels of acculturative stress compared to men and are more prone to experience depressive symptoms.

In our study, the year of study was negatively related to homesickness and stress due to change/culture shock. Also, age was negatively correlated with some subscales of the ASSIS: homesickness, stress due to change/culture shock, guilt, and nonspecific concerns. The research on this association is not yet conclusive. Some studies found no association between age and acculturative stress [22, 37]. However, Ye [38] found higher levels of acculturative stress among older Chinese international students compared to younger ones. The results of that study showed that older students scored higher on fear, perceived discrimination, and hatred than younger students. Also, Akhtar and Kröner-Herwig [34] found that younger age predicted a low level of acculturative stress. However, those studies were not conducted on medical students. Given the fact that medical studies span over a period of six years, it might be plausible for some aspects of the acculturation process to differ from other studies.

It has been previously documented that international students tend to experience language difficulties [39] and the challenges posed by them might make it difficult to interact with their peers [40]. In the present study, the degree of comfortability of speaking in English correlated negatively with the ASSIS and all its subscales. Also, the more comfortable the students feel communicating in Romanian, the less they score on the stress due to change/culture shock subscale of the ASSIS. Accordingly, before taking into consideration the option of studying abroad, students should focus on attaining language proficiency. This allows increased chances of social interaction with the members of the host society and could be an important factor in decreasing stressful experiences [34].

Studying the acculturative stress among international students from different specialties, Sullivan and Kashubeck-West [41] assert that home country support and an emphasis on maintaining ties to the home culture are not beneficially associated with adapting to studies in the United States. In our study, having origins from the host country seemed to be an important factor that decreased the level of stress among international students. More specifically, students who had Romanian origins had lower scores on perceived hate and stress due to change/culture shock compared to international students with no Romanian origins.

Our study also indicated that friends and family presence decreased the rate of acculturative stress, perceived discrimination, perceived hate/rejection, and nonspecific concerns and might indicate their influence on the choice to study in a certain country. Students speaking Romanian or having one parent speaking Romanian had lower levels of stress due to change/culture shock. Finally, students who were not visited by their parents every year were more prone to experience homesickness. International students with Romanian origins conserved a sort of host-country contact by parents or grandparents (language, food, relatives, family members etc); therefore, the host-culture is not likely entirely new to them and their level of stress is low. Previous studies [42, 43] underlined the fact that acculturative stress might be decreased by the development of host country rather than home country social support. The results of our study showed that, although students still have ties with their home country (through the presence of family and friends at the same University and visits from their parents), they also have stable connections with the host country (through their origins and the fact that either them or at least one of their parents spoke Romanian), and these aspects contribute to a low level of acculturative stress. However, this result is specific to our sample (given the fact that close to half of our participants had friends or family enrolled in the same university) and might be due to the ways students choose their university [1, 2].

Students who decide to study in another country might experience practical or lifestyle acculturative stressors. When transitioning from their home country to their host country, international college students face various perceived threats and challenges. Among them are the lack of knowledge of the host culture, difficulty in adapting to the host country customs and lifestyle, and maladjustment to the physical environment [44]. The present study took into consideration some of these aspects: food, climate, transportation, neighbors, and communication with the homeowner. The results showed negative correlations between these variables and acculturative stress and some of its subscales. The students' transition from their country to the country where they will study brings the need to adopt and assimilate the customs and culture of their host country. Adjusting to food, weather, accommodation, and local language represents an important challenge for international students [26, 45]. Ideally, international students should gather information regarding geographical and social aspects, food, and transportation in their targeted country. However, documentation does not provide the same experience as personal experience and the students must be aware of this aspect.

International student satisfaction is an in important factor in strengthening support services for this community [46]. Previous studies have found that students struggled to develop friendships with local and international students and often felt disconnected with the wider campus community outside the classroom [47]. Our study showed that higher levels of satisfaction regarding the relationship with colleagues, teachers, and administrative staff were related to lower levels of acculturative stress (and most of the subscales of the ASSIS). Baranova et al. [48] found that two of the main factors contributing to improved student experience are increased customer service training for service staff and a revitalized program that should focus on students' transition and acculturation to their new campus environment. The authors also mention that, in the case of countries with tradition in international recruitment, in order to draw and keep international students, universities must offer them suitable support services and resources; in order to be competitive in this area, there is a need to improve the international student experience [49].

### 4.1. Reflections and Planning

The drop-out rates in this medical university is about 10%, according to the rates identified by the university career counselling centre. The most important reason declared by the students being the financial one even if the level of annual taxes for medical studies in Romania are among the lowest in Europe.

The results of the present research must encourage teaching and counselling staff to adopt more diversified and efficient strategies for helping international students to cope with acculturative stress: establishing a more developed mentoring program for freshmen students matching them with seniors, for advice and support; increasing the number of intercultural events in order to help students socialize; providing more flexible support services in order to help them adjust to the new academic demands and cultural/local habits); organizing meetings with national and international students for networking and facilitating information change regarding lifestyle, local transportation, facilities for sports, leisure, or hobbies; and enhancing counsellors and therapist awareness in working with international students and providing them with information and knowledge on how to work with multicultural and multilingual groups.

### 4.2. Strengths and Limitations of the Study

The research on acculturation and acculturative stress among international medical students is scarce, and the present study provides some insights into this population. Because women are more numerous than men in medical universities, most studies provided results for a smaller number of female students than males. The present study included a balanced gender distribution.

This study has several limitations. The research did not evaluate the mental health of students. The authors know the presence of depression, anxiety, or chronic diseases could increase the rate of acculturative stress. Also, we used a convenience sample from a single medical university. Further research should be done with different sampling methods. Our study was correlational, which does not prove causal relations between the variables. Also, the use of the English language is always a concern with international populations, and it raises the question of whether participants' language skills are adequate to appropriately answer the survey. Considering these limitations, findings should be generalized with caution.

## 5. Conclusions

The existence of risk factors for acculturative stress demands institutional, social, and psychological support for international students. Multicultural environments must be provided with resources to maintain a sustainability development of international students during their process of education. Apart from their psychological characteristics and inner motivation, the support from family members, peers, academic community, social media, or professional staff working in the university field can also provide support for students during their academic trajectory and career.

## Figures and Tables

**Table 1:** Sociodemographic characteristic and family-related data.

Variables		%
Sex	Male	57
	Female	43
Year of study	1^st^	44.9
	2^nd^	13.2
	3^rd^	17.4
	4^th^	6.4
	5^th^	6.8
	6^th^	11.3
Age (*M* ± SD)		21.38 (3.32)
Romanian origins		9.1
Parent physician		22.6
Friends or other relatives enrolled in the same university		42.3
Student speaks Romanian		7.9
Mother speaks Romanian		9.8
Father speaks Romanian		13.6
Parents visit every year		35.1
Satisfied with living conditions		69.1

^∗^Percentages (%), means (*M*), and standard deviations (SD).

**Table 2:** The distribution of answers to items regarding students' comfortability with some aspects of life in the university City.

How comfortable are you with…	Not comfortable	Somewhat comfortable	Comfortable	Very comfortable	Extremely comfortable	*M* (SD)
Speaking Romanian	35.8	34.0	18.1	7.5	4.5	2.10 (1.11)
Speaking English	2.6	4.9	25.3	35.8	31.3	3.88 (0.99)
Country's climate	12.1	24.2	40.8	18.5	4.5	2.79 (1.02)
City transportation	17.0	26.0	38.1	15.8	3.0	2.61 (1.03)
Neighbors	12.1	15.5	41.9	20.8	9.8	3.00 (1.11)
Local food	15.5	26.8	37.0	10.2	10.6	2.73 (1.16)
Communicating with the homeowner	6.4	15.1	39.2	21.9	17.4	3.28 (1.16)

^∗^Percentages (%), means (M) and standard deviations (SD).

**Table 3:** Total score and scores for each subscale of ASSIS.

Total score and subscales	Results
ASSIS (total score)	85.82 (23.03)
Perceived discrimination	18.95 (5.91)
Homesickness	11.35 (3.40)
Perceived hate/rejection	11.64 (3.97)
Fear	8.74 (3.09)
Stress due to change/culture shock	7.52 (2.44)
Guilt	4.77 (1.77)
Nonspecific concerns	22.83 (6.96)

^∗^Means (*M*) and standard deviations (SD).

**Table 4:** Correlations between ASSIS (total score and subscores) and variables.

	ASSIS	Perceived discrimination	Homesickness	Perceived hate/rejection	Fear	Stress due to change/culture shock	Guilt	Non-specific concerns
Age	*r* = −0.170 *p* = 0.005	*r* = 0.078 *p* = 0.207	*r* = −0.307 *p* = 0.000	*r* = −0.077 *p* = 0.213	*r* = −0.119 *p* = 0.052	*r* = −0.221 *p* = 0.000	*r* = −0.174 *p* = 0.005	*r* = −0.128 *p* = 0.037
Year of study	*r* = −0.118 *p* = 0.055	*r* = −0.035 *p* = 0.566	*r* = −0.364 *p* = 0.000	*r* = 0.002 *p* = 0.971	*r* = −0.030 *p* = 0.630	*r* = −0.165 *p* = 0.007	*r* = −0.130 *p* = 0.034	*r* = −0.075 *p* = 0.225
Comfortable speaking Romanian	*r* = −0.092 *p* = 0.137	*r* = −0.075 *p* = 0.222	*r* = −0.095 *p* = 0.124	*r* = −0.086 *p* = 0.165	*r* = −0.106 *p* = 0.085	*r* = −0.150 *p* = 0.014	*r* = −0.014 *p* = 0.819	*r* = −0.047 *p* = 0.448
Comfortable speaking English	*r* = −0.293 *p* = 0.000	*r* = −0.215 *p* = 0.000	*r* = −0.172 *p* = 0.005	*r* = −0.250 *p* = 0.000	*r* = −0.237 *p* = 0.000	*r* = −0.238 *p* = 0.000	*r* = −0.288 *p* = 0.000	*r* = −0.316 *p* = 0.000
Comfortable with country's climate	*r* = −0.233 *p* = 0.000	*r* = −0.205 *p* = 0.001	*r* = −0.166 *p* = 0.007	*r* = −0.190 *p* = 0.002	*r* = −0.186 *p* = 0.002	*r* = −0.199 *p* = 0.001	*r* = −0.152 *p* = 0.013	*r* = −0.184 *p* = 0.003
Comfortable with local transport	*r* = −0.099 *p* = 0.108	*r* = −0.130 *p* = 0.035	*r* = 0.067 *p* = 0.277	*r* = −0.120 *p* = 0.051	*r* = −0.073 *p* = 0.239	*r* = −0.071 *p* = 0.252	*r* = −0.017 *p* = 0.780	*r* = −0.099 *p* = 0.107
Comfortable with neighbors	*r* = −0.246 *p* = 0.000	*r* = −0.287 *p* = 0.000	*r* = −0.068 *p* = 0.267	*r* = −0.186 *p* = 0.002	*r* = −0.233 *p* = 0.000	*r* = −0.242 *p* = 0.000	*r* = −0.111 *p* = 0.070	*r* = −0.277 *p* = 0.000
Comfortable with local food	r=−0.240 p=0.000	r=−0.185 p=0.003	r=−0.114 p=0.064	r=−0.210 p=0.001	r=−0.243 p=0.000	r=−0.328 p=0.000	r=−0.188 p=0.002	r=−0.205 p=0.001
Comfortable communicating with homeowner	r=−0.126 p=0.040	r=−0.131 p=0.033	r=0.053 p=0.390	r=−0.105 p=0.089	r=−0.166 p=0.007	r=−0.108 p=0.078	r=−0.015 p=0.804	r=−0.163 p=0.008
Satisfaction with colleagues	r=−0.266 p=0.000	r=−0.222 p=0.000	r=−0.155 p=0.012	r=−0.244 p=0.000	r=−0.221 p=0.000	r=−0.242 p=0.000	r=−0.135 p=0.028	r=−0.232 p=0.000
Satisfaction with teachers	r=−0.258 p=0.000	r=−0.265 p=0.000	r=−0.007 p=0.916	r=−0.220 p=0.000	r=−0.300 p=0.000	r=−0.257 p=0.000	r=−0.141 p=0.022	r=−0.234 p=0.000
Satisfaction with administrative staff	r=−0.275 p=0.000	r=−0.294 p=0.000	r=−0.047 p=0.445	r=−0.218 p=0.000	r=−0.271 p=0.000	r=−0.152 p=0.013	r=−0.169 p=0.006	r=−0.275 p=0.000

^∗^A *p* value lower than 0.05 was considered statistically significant.

## Data Availability

The data used to support the findings of this study are available from the corresponding author upon request.

## Supplementary Materials

Supplementary Materials Table 5: independent samples *t*-tests and Benjamini-Hochberg procedure.
