# Supplementary material for: Factors Associated with Acculturative Stress among International Medical Students
Source: Biomed Res Int. 2020 Jun 21;2020:2564725. doi: 10.1155/2020/2564725 (PMC7327593; doi:10.1155/2020/2564725)
Supplement: Supplementary Materials — Table 5: independent samples t-tests and Benjamini-Hochberg procedure. [file 2564725.f1.docx]

| Table 5. Independent samples t-tests and Benjamini-Hochberg procedure | | | | |
| --- | --- | --- | --- | --- |
| Independent variable | Dependent variable | p-value | Rank | (i/m)Qª |
| Gender (male/female) | ASSIS (*total score*) | .074 | 16 | .062 |
|  | Perceived discrimination | .729 | 50 | .195 |
|  | **Homesickness** | **.000** | **1** | **.003** |
|  | Perceived hate/rejection | .359 | 38 | .148 |
|  | Fear | .340 | 34 | .132 |
|  | **Stress due to change/cultural shock** | **.036** | **10** | **.039** |
|  | Guilt | .201 | 28 | .109 |
|  | Non-specific concerns | .299 | 33 | .128 |
| Romanian origins (yes/no) | ASSIS (*total score*) | .064 | 13 | .050 |
|  | Perceived discrimination | .186 | 25 | .097 |
|  | Homesickness | .489 | 44 | .171 |
|  | **Perceived hate/rejection** | **.036** | **10** | **.039** |
|  | Fear | .108 | 19 | .074 |
|  | **Stress due to change/cultural shock** | **.003** | **2** | **.007** |
|  | Guilt | .344 | 36 | .140 |
|  | Non-specific concerns | .146 | 22 | .085 |
| Parent doctor (yes/no) | ASSIS (*total score*) | .389 | 39 | .152 |
|  | Perceived discrimination | .942 | 57 | .222 |
|  | **Homesickness** | **.031** | **7** | **.027** |
|  | Perceived hate/rejection | .933 | 56 | .218 |
|  | Fear | .856 | 55 | .214 |
|  | Stress due to change/cultural shock | .531 | 45 | .175 |
|  | **Guilt** | **.003** | **2** | **.007** |
|  | Non-specific concerns | .357 | 37 | .144 |
| Relatives enrolled at the | **ASSIS (*total score*)** | **.040** | **11** | **.042** |
| same university (yes/no) | **Perceived discrimination** | **.022** | **6** | **.023** |
|  | Homesickness | .991 | 59 | .230 |
|  | **Perceived hate/rejection** | **.020** | **5** | **.019** |
|  | Fear | .066 | 14 | .054 |
|  | Stress due to change/cultural shock | .269 | 32 | .125 |
|  | Guilt | .262 | 31 | .121 |
|  | **Non-specific concerns** | **.043** | **12** | **.046** |
| Student speaks Romanian | ASSIS (*total score*) | .176 | 23 | .089 |
| (yes/no) | Perceived discrimination | .399 | 40 | .156 |
|  | Homesickness | .671 | 48 | .187 |
|  | Perceived hate/rejection | .129 | 21 | .082 |
|  | Fear | .095 | 18 | .070 |
|  | **Stress due to change/cultural shock** | **.000** | **1** | **.003** |
|  | Guilt | .420 | 41 | .160 |
|  | Non-specific concerns | .570 | 46 | .179 |
| Mother speaks Romanian | **ASSIS (*total score*)** | **.032** | **8** | **.031** |
| (yes/no) | Perceived discrimination | .240 | 30 | .117 |
|  | Homesickness | .180 | 24 | .093 |
|  | **Perceived hate/rejection** | **.034** | **9** | **.035** |
|  | Fear | .067 | 15 | .058 |
|  | **Stress due to change/cultural shock** | **.000** | **1** | **.003** |
|  | Guilt | .077 | 16 | .062 |
|  | Non-specific concerns | .082 | 17 | .066 |
| Father speaks Romanian | ASSIS (*total score*) | .198 | 27 | .105 |
| (yes/no) | Perceived discrimination | .667 | 47 | .183 |
|  | Homesickness | .474 | 43 | .167 |
|  | Perceived hate/rejection | .112 | 20 | .078 |
|  | Fear | .226 | 29 | .113 |
|  | **Stress due to change/cultural shock** | **.019** | **4** | **.015** |
|  | Guilt | .191 | 26 | .101 |
|  | Non-specific concerns | .343 | 35 | .136 |
| Parents visit every year | ASSIS (*total score*) | .832 | 54 | .210 |
| (yes/no) | Perceived discrimination | .429 | 42 | .164 |
|  | **Homesickness** | **.018** | **3** | **.011** |
|  | Perceived hate/rejection | .944 | 58 | .226 |
|  | Fear | .756 | 51 | .199 |
|  | Stress due to change/cultural shock | .825 | 53 | .207 |
|  | Guilt | .681 | 49 | .191 |
|  | Non-specific concerns | .814 | 52 | .203 |
| ªCorrected p-value using the Benjamini-Hochberg procedure: i is the rank, m is the total number of tests (64), and Q is the false discovery rate (25%). | | | | |
